# Ruthenium nanoparticles stabilized by mercaptan and acetylene derivatives with supercapacitor application

**DOI:** 10.1016/j.mex.2018.07.004

**Published:** 2018-07-07

**Authors:** Yan Guo, Wanying Zhang, Yang Sun, Mingming Dai

**Affiliations:** a Collaborative Innovation Center of Atmospheric Environment and Equipment Technology, School of Environmental Science and Engineering, Nanjing University of Information Science and Technology, Nanjing, Jiangsu 210044, China; b Reading Academy, Nanjing University of Information Science and Technology, Nanjing, Jiangsu 210044, China

**Keywords:** Hydrazine hydrate reduction, Ruthenium nanoparticles, Organic ligand, Supercapacitor, Cyclic voltammetry, Charge-discharge analysis

## Abstract

Ruthenium nanoparticles (RuHT, RuPET and RuPA) were prepared by hydrazine hydrate reduction of RuCl_3_ and stabilized by the self-assembly of organic molecules (hexanethiol, phenylethanethiol and phenylacetylene). The sizes of these Ru nanoparticles were carried out by transmission electron microscopic measurement, with the average core sizes of 2.84 ± 0.55 nm, 3.06 ± 1.22 nm, and 3.10 ± 1.08 nm, respectively. The structures and properties of these Ru nanoparticles were further examined and verified by UV–vis, FTIR, ^1^HNMR, XPS and fluorescent measurements. The performance of the supercapacitor was characterized by cyclic voltammetry and constant-current charge-discharge analysis. Ru nanoparticles exhibited enhanced supercapacitor behaviors as compared with blank electrodes. The Ru nanoparticles for supercapacitors in the H_2_SO_4_ electrolyte exhibited areal capacitances of 347.8, 304.9 and 229.1 mF cm^−2^ for RuPET, RuPA and RuHT at a scan rate of 10 mV s^−1^, and specific capacitances for 344.4, 249.3, 230.0 F g^−1^ for RuPET, RuPA and RuHT at a current density of 0.5 A g^−1^, respectively. The interfacial bonding between ruthenium and the outlayer organic ligands and varied ratio of ruthenium in high valence might be the reasonable explanation for the capacitance difference.

## Graphical abstract

**Figure:**
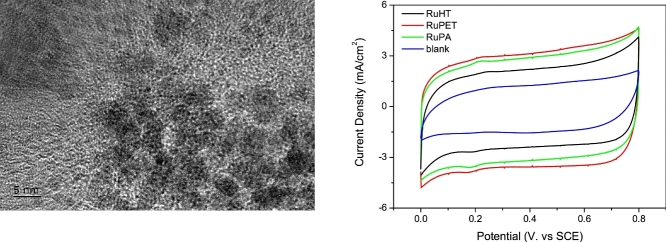

• Ru nanoparticles were protected by organic ligands via a simple reduction method.
• These Ru nano-compounds were almost the same core size by this method.
• The properties of Ru nanoparticles could be easily controlled.

**Specifications Table**

**Table:** 

Subject area	Select one of the following subject areas:
	• Agricultural and Biological Sciences • Biochemistry, Genetics and Molecular Biology • Chemical Engineering • Chemistry • Computer Science • Earth and Planetary Sciences • Energy • Engineering • Environmental Science • Immunology and Microbiology • Materials Science • Mathematics • Medicine and Dentistry • Neuroscience • Pharmacology, Toxicology and Pharmaceutical Science • Physics and Astronomy • Psychology • Social Sciences • Veterinary Science and Veterinary Medicine
More specific subject area	Analytical Chemistry
Method name	hydrazine hydrate reduction
Name and reference of original method	1 X. Kang, N. B. Zuckerman, J. P. Konopelski, S. Chen, Alkyne-functionalized ruthenium nanoparticles: ruthenium-vinylidene bonds at the metal-ligand interface, J. Am. Chem. Soc. 134 (2012) 1412. 2 Y. Guo, L. Chen, Y. Song, P. Hu, S. Chen, Sci. Ruthenium nanoparticles stabilized by the self-assembly of acetylene, carboxylate, and thiol derivatives, Adv. Mater. 6 (2014) 1060.
Resource availability	1 http://pubs.acs.org/doi/pdfplus/10.1021/ja209568v 2 http://www.ingentaconnect.com/content/asp/sam/2014/00000006/00000005/art00024

## Method details

The Ru nanoparticles were prepared by hydrazine hydrate reduction method, similar procedure that has been used previously in our work [1,2]. In a typical reaction, for example, approximate 20.8 mg RuCl_3_ (0.1 mmol) was dissolved in about 1 mL ethanol. Then, the dark wine solution was transferred to 10 ml toluene solution. Then, 32 μL phenylacetylene (or 40 μL 2-phenylethanethiol or 42 μL 1-hexanethiol) was added, and let these ligands contact with Ru ions completely for 30 min. 8 mg hydrazine hydrate (about 2 fold excess equivalents) was used and added drop by drop with a dropping pipet. After the addition of hydrazine hydrate, the reaction was allowed to stir for 24 h to prepare organic-capped Ru nanoparticles. After the reaction, organic solvent was then removed by rotary evaporator. The resulting sample was then washed several times with acetonitrile and ethanol, successively to remove excess ligands. Finally, the purified Ru nanoparticles were dissolved and kept in dichloromethane (DCM), and the solution presents dark brown or black color.
